# Testing conditional multivariate rank correlations: the effect of institutional quality on factors influencing competitiveness

**DOI:** 10.1007/s11749-022-00806-1

**Published:** 2022-04-01

**Authors:** Jone Ascorbebeitia, Eva Ferreira, Susan Orbe

**Affiliations:** Department of Quantitative Methods, University of the Basque Country UPV/EHU, Avda. Lehendakari Aguirre 83, 48015 Bilbao, Spain

**Keywords:** Conditional copula, Nonparametric estimation, Multivariate dependence, Kendall’s tau, 62H05 Copulas, 62G05 Nonparametric estimation, 62H20 Measures of association, 62G15 Hypothesis testing in multivariate analysis

## Abstract

Joint distribution between two or more variables could be influenced by the outcome of a conditioning variable. In this paper, we propose a flexible Wald-type statistic to test for such influence. The test is based on a conditioned multivariate Kendall’s tau nonparametric estimator. The asymptotic properties of the test statistic are established under different null hypotheses to be tested for, such as conditional independence or testing for constant conditional dependence. Two simulation studies are presented: The first shows that the estimator proposed and the bandwidth selection procedure perform well. The second presents different bivariate and multivariate models to check the size and power of the test and runs comparisons with previous proposals when appropriate. The results support the contention that the test is accurate even in complex situations and that its computational cost is low. As an empirical application, we study the dependence between some pillars of European Regional Competitiveness when conditioned on the quality of regional institutions. We find interesting results, such as weaker links between innovation and higher education in regions with lower institutional quality.

**Supplementary Information:**

The online version contains supplementary material available at 10.1007/s11749-022-00806-1.

## Introduction

The joint distribution of two or more variables is a topic of clear interest in statistics. Actually, it provides a wealth information to analyze the degree of dependence and the effects of conditional variables on comovements, among others. A classic measure of the degree of dependence is pairwise linear correlation. For a multivariate setting, the average of the pairwise linear correlations is widely used as a measure of multivariate dependence (see for example Joe (1990), Longin and Solnik (1995), Moskowitz (2003), Capiello et al. (2006), Pollet and Wilson (2010)).

Beyond Pearson’s correlation and normal distributions, Kendall’s tau provides a descriptive statistic that detects monotonicity rather than linearity and has the benefit of not being restricted to symmetric distributions. For a multivariate context, Kendall and Smith (1940) suggest the average of pairwise taus as a coefficient of agreement. Another approach is followed by Simon (1977), who suggests a sign function-based multivariate extension. In a related paper, Joe (1989) finds that the latter proposal does not meet the properties for consideration as a concordance measure. Therefore, Joe (1990) suggests a family of unconditional measures based on Kendall’s tau as multivariate concordance measures beyond the average of pairwise taus. In this line, Nelsen (1992) and Nelsen (1996) analyze Kendall’s tau-based bivariate and multivariate measures.

Our first aim is to estimate a conditional multivariate Kendall’s tau to analyze the effect of a variable of interest in the strength of dependence. To that end, we extend the proposal made by Joe (1990) to the conditional case and define a multivariate conditional Kendall’s tau estimator as an extension of Gijbels et al. (2011), with the corresponding asymptotic results.

Bandwidth selection is an important practical issue in nonparametric estimation. In this sense, Silverman (1986) is a well-known reference for nonparametric densities as well as Altman and Leger (1995), Sarda (1993), and Bowman et al. (1998) for nonparametric distributions, who propose plug-in and cross-validation methods. In order to select the bandwidth for the nonparametric Kendall’s tau estimator, we propose to minimize its mean squared error based on plug-in steps as in Gijbels et al. (2011). Bouezmarni et al. (2019) also use a similar recursive procedure of bandwidth selection for nonparametric local causality measures. As a data-driven method, we provide a jackknife method for estimating bias and variance. We also derive a simulation study to show the good performance of the multivariate estimator and the bandwidth selection procedure in practice.

The second aim of the methodological part of our study is to provide a statistic to test for the structure of dependence. For unconditional multivariate independence, there are well-known tests in the literature [see e.g., (Genest and Rémillard 2004)]. When variables are normally distributed, a test based on linear correlation is suitable. For more general cases, Leung et al. (2018) propose a rank correlation-based test for independence between variables in high dimensions. In a related paper, Mao (2018) proposes a new test with better performance for large and small samples that deals with size distortion problems detected in the proposal of Leung et al. (2018) for small sample sizes. Strzalkowska-Kominiak and Stute (2013) also propose rank correlation-based statistics to test for independence with survival data.

Here, we are interested in testing for conditional dependences, and in particular for general restrictions among conditional Kendall’s taus. In a related paper, Gijbels et al. (2017) analyze the so-called simplifying assumption, which assumes that the conditional copula coincides with the partial copula. To do so, they propose tests expressed as a linear restriction between conditional Kendall’s taus and compare their performance with other tests based on conditional copulas, such as the test proposed by Acar et al. (2013). Using another approach, Bouezmarni et al. (2019) define Kendall’s tau causality measures based on local causality copulas and propose tests for local non-causality based on such measures.

Our proposal is to derive a Wald-type statistic to test for general linear restrictions. The statistic enables tests to be run for specific, interesting cases such as constant conditional dependence, linear restrictions between different Kendall’s taus, and equality of conditional Kendall’s tau across different populations.

To study its performance in practice, we run a simulation study and compare the results with the proposal in Gijbels et al. (2017) for those situations where their test can be applied. Results support the idea that the Wald-type test performs better for many different models, with a noteworthy advantage in computational cost.

For an empirical application, we consider the European Union Regional Competitiveness Index (EU-RCI) data for 2019 for 268 regions. The index is formed by eleven pillars that help to identify the strengths and weaknesses in competitiveness of each region. All pillars provide indicators for the prosperity of a region and they are linked to one another. For instance, the more prosperous a region is, the better the indicators for all groups are expected to be. Obviously, this relationship is not perfect, and that is one reason to draw up a combined index. The overall index will reflect this combination of pillars. A classic question of interest is whether changes in one pillar are related to changes in another. Here, another question arises: How the value of one pillar can influence or is related to the intensity of cross relations between other pillars. The test proposed is used to test for any significant effect of one pillar on the relationship between others.

In particular, we focus on the quality of regional institutions, which plays a very important role in the prosperity of a region. Dimant and Tosato (2018) provide a very helpful review of empirical results from the past few decades. One of the pillars in the Basic group is the Institutions index, which reports the quality of governments at regional and national levels, measured through perception and experience as collected via a survey. The interest lies in detecting the contribution of institutional quality to the relationship between pillars and in testing for changes in those relationships conditional on institutional quality levels. Moreover, tests for any other type of restriction can be run, such as changes in effects across different waves.

We find that tests of this type have interesting applications in practice in many different fields, particularly in medicine. For instance, Echouffo-Tcheugui et al. (2018) find that higher cortisol levels are associated with worse memory and visual perception. Moreover, morning rises in cortisol levels have been found to increase with body mass index. In this context, it would be interesting to test whether the relationship between cortisol as a “stress hormone” and the cognitive performance of patients changes with body mass index. Related to the current COVID-19 pandemic, Toyoshima et al. (2020) analyze individuals mutations in SARS-CoV-2 genome sequences and their relationship with fatality rates, concluding that some virus variants are significantly correlated with them. Since host differences contribute to variations in response to pathogens, this test enables the effects of factors such as the genetics, age, and obesity of patients to be assessed in the relationships measured by the rank correlation to better understand the spread of COVID-19 and improve vaccine efficacy.

The rest of the paper is structured as follows. Section 2 states the main results for the nonparametric estimators and sets out the practical estimation and testing procedures. Section 3 provides the simulation studies that show the performance of the smoothing parameter selection and of the test proposed. Section 4 applies the methodology to the link between regional efficiency and innovation pillars conditional on the quality of institutions. Section 5 concludes. Details of proofs and additional simulation results are given as supplementary material.

## Estimation and tests

Let $\varvec{Y}=\{Y_j\}_{j=1}^p$ be a set of *p* variables, and $F_{1},\ldots , F_{p}$ and *F* their continuous marginal and joint distributions, respectively. In this context, Sklar (1959) states that there is a unique copula function $C\!: [0,1]^p \rightarrow [0,1]$ such that $F(\varvec{y})=F(y_{1},\ldots , y_{p})=C(F_{1}(y_{1}),\ldots , F_{p}(y_{p}))$ for all $\varvec{y}=(y_1,\ldots , y_p)\in \varvec{\mathbb {R}}^p$. That is, copulas are joint distribution functions whose marginals are standard uniform variables. Patton (2006) extends this result to conditional copulas and states that, given a covariate *Z*, there is a unique copula $C_z:[0,1]^p\rightarrow [0,1]$ such that $F_z(\varvec{y})=C_z(F_{1z}(y_{1}),\ldots , F_{pz}(y_{p}))$, where $F_{jz}(y)=P(Y_j\le y|Z=z)$, for any $y\in Y_j$, $j=1,\ldots ,p$. In inverting Sklar’s theorem, the $C_z$ function can be expressed as $C_z(\varvec{u})=F_z(F_{1z}^{-1}(u_{1}),\ldots , F_{pz}^{-1}(u_p))$ in terms of the joint and marginal distribution functions, where $\varvec{u}=(u_1,\ldots , u_p)\in [0,1]^p$ and $F_{jz}^{-1}(u)=inf\{y: F_{jz}(y)\ge u\}$ is the *z*-conditional quantile function of $Y_j$.

To estimate conditional copulas, Gijbels et al. (2011) propose a nonparametric estimator in a bivariate context. We use the natural extension to the multivariate conditional copula estimator,

1 $$\begin{aligned} \hat{C}_{z,h_n}(\varvec{u})= & {} \sum _{i=1}^n w_i(z, h_n)I \left\{ Y_{1i}\le \hat{F}_{1z,h_n}^{-1}(u_1),\ldots , Y_{pi}\le \hat{F}_{pz,h_n}^{-1}(u_p)\right\} , \end{aligned}$$

where $\{w_i(z, h_n)\}$ is a sequence of weights depending on $(z-Z_i)/h_n$ and $h_n$ is the bandwidth. Considering Nadaraya–Watson weights, $\{w_i(z, h_n)\} = k((z-Z_i)/h_n)/\sum _j k((z-Z_j)/h_n)$, where *k* is a kernel function. $I\{\cdot \}$ is the indicator function and $\hat{F}_{jz,h_n}(y)=\sum _{i=1}^n w_i(z, h_n)I\{Y_{ji}\le y\}$ is the nonparametric conditional *j*-marginal estimator. It is noteworthy that the bandwidth in this case does not have the usual smoothing effect as in regression. In fact, when the bandwidth $h_n$ increases, the copula estimator $\hat{C}_{z,h_n}$ tends to the empirical copula $\hat{C}_{z}(\varvec{u})=n^{-1}\sum _{i=1}^n I\{Y_{1i}\le \hat{F}_{1z}^{-1}(u_1),\ldots , Y_{pi}\le \hat{F}_{pz}^{-1}(u_p)\}$.

To quantify the degree of dependence, we estimate the Kendall’s tau coefficient as a measure of the ordinal association between two measured quantiles. The multivariate Kendall’s tau is defined as in Joe (1990), $\tau =(2^{p-1}-1)^{-1}\left( 2^p \int _{\mathbf {I}^p} C(\varvec{u})dC(\varvec{u})-1\right) ,$ where $\mathbf {I}^p=[0,1]^p$. The multivariate Kendall’s tau accounts for common comovements beyond pairwise effects and quantifies simultaneous concordance. Thus, more variables imply more conditions to be met at the same time and so fewer concordances are expected. The distortion that the number of variables can produce is mitigated by the *p*-dependent correction factor included in the definition of the tau. Note that Kendall’s tau is a measure of dependence that depends only on the copula and not on the marginals. We also note the advantage of the multivariate Kendall’s tau over the pairwise average as an overall dependence measure, since it accounts for multivariate distribution and not only for bivariate effects. As the multivariate nonparametric estimator of $\tau$, we consider an extended version of the empirical bivariate Kendall’s tau (Deheuvels 1980),

$$\begin{aligned} \hat{\tau }=\frac{1}{2^{p-1}-1}\Big (\frac{2^p}{n(n-1)}\sum _{i=1}^n \sum _{j=1}^n I\{\mathbf {Y}_{i}\!<\!\mathbf {Y}_{j}\}-1\Big ), \end{aligned}$$

where $$\varvec{Y}_{i}=(Y_{1i},\ldots ,Y_{pi})$$ and $$I\{\mathbf {Y}_{i}<\mathbf {Y}_{j}\}=I\{Y_{1i}<Y_{1j},\ldots ,Y_{pi}<Y_{pj}\}$$.

An extended version of the multivariate Kendall’s tau proposed by Joe (1990) to conditional copulas can be defined as

2 $$\begin{aligned} \tau _{z}=\frac{1}{(2^{p-1}-1)}\left( 2^p \int _{\mathbf {I}^p} C_z(\varvec{u})dC_z(\varvec{u})-1\right) . \end{aligned}$$

The nonparametric estimator proposed is

3 $$\begin{aligned} \hat{\tau }_{z,h_n}\!=\!\frac{1}{2^{p\!-\!1}\!\!-\!1}\left( \!\frac{2^p}{1\!-\!\!\sum _{i=1}^n\!\! w_i(z, h_n)^2}\!\!\sum _{i,j=1}^n \!\! w_i(z, h_n)w_j(z, h_n)I\{\mathbf {Y}_{i}\!<\!\mathbf {Y}_{j}\}\!-\!1\!\right) , \end{aligned}$$

where the weights are based on the recommendations given by Gijbels et al. (2011). Note that as for the copula estimator, the conditional Kendall’s tau (3) tends to the unconditional empirical Kendall’s tau as the bandwidth increases. This estimator generalizes the bivariate estimator in Gijbels et al. (2011). The asymptotic normality of the conditional Kendall’s tau estimator is established by Veraverbeke et al. (2011) for the bivariate case. The next proposition generalizes the consistency and asymptotic normality of the multivariate conditional Kendall’s tau estimator in (3) under the usual set of assumptions:

1. $$(\varvec{Y}_{i},Z_{i})$$, $$i=1,\ldots ,n$$ are *i*.*i*.*d*. tuples.
2. The conditional joint distribution $F_z(\cdot )=F(\cdot |z)$ and the density of the covariable *Z*, *f*(*z*), have continuous first and second order derivatives with respect to *z*, all denoted with the respective primes.
3. The kernel is a bounded symmetric second-order kernel with compact support $\Omega =[-1,1]$ such that $\int _{\Omega }k(\eta )d\eta =1$. Moreover, $c_k=\int _{\Omega } k(\eta )\eta ^2d\eta$ and $d_k=\int _{\Omega } k(\eta )^2d\eta$ are nonzero quantities.
4. $$h_n\rightarrow 0$$ and $$nh_n\rightarrow \infty $$ as $$n\rightarrow \infty $$.

### Proposition 1

Under assumptions A1 to A4, the conditional Kendall’s tau estimator $\hat{\tau }_{z,h_n}$ defined in (3) is a consistent estimator of $\tau _z$ defined in (2), where the asymptotic bias is $Bias(\hat{\tau }_{z,h_n})=2^{p\!-\!1}h_n^2c_k((2^{p\!-\!1}\!-\!1)\!f(z))^{-1}\!\!\int _{\mathbb {R}^p}\!\! \Big (\!F_z(\varvec{y})\times$ $g\left( f_z(\varvec{y})\right) +f_z(\varvec{y})g\left( F_z(\varvec{y})\right) \Big ) d\varvec{y}+o(h_n^2)$ with $g(r_{z}(\varvec{y}))=\Big (\!r_{z}(\varvec{y})f''(z)+2r'_{z}(\varvec{y})f'(z)+r''_{z}(\varvec{y})f(z)\Big )$.

Moreover, if $C_z^L$ is the limiting distribution of $\left( nh_n\right) ^{1/2}\big (\hat{C}_{z,h_n}(\varvec{u})-C_z(\varvec{u})\big )$ and $\varphi _z$ is a Gaussian variable given by

$$\begin{aligned} \varphi _z=2^p(2^{p-1}\!-\!1)^{-1}\left( \int _{I^p}C_z(\varvec{u})dC_z^L(\varvec{u})+\int _{I^p}C_z^L(\varvec{u})dC_z(\varvec{u})\right) , \end{aligned}$$

the asymptotic variance of $\hat{\tau }_{z,h_n}$ is given by the variance of $\varphi _z$, $\sigma ^2(\varphi _z)$. Additionally, assuming that $h_n=o(n^{-1/5})$ and $\int _{\Omega }k(\eta )^{\zeta }d\eta \ne 0$ for $\zeta >2$,

$$\begin{aligned} \left( nh_n\right) ^{1/2}\left( \hat{\tau }_{z,h_n}-\tau _z\right) \xrightarrow {d}\varphi _z. \end{aligned}$$

The limiting distribution of the multivariate estimator (3) is obtained from the asymptotic normality of the conditional copula estimator $$\hat{C}_{z,h_n}(\varvec{u})$$, provided that Kendall’s tau can be written as a functional of the copula and the Hadamard differentiability of such functional (tangentially to the set of continuous functions on $$[0,1]^p)$$. The details are given in Appendix A.

### Bandwidth selection

Classic proposals for selecting the smoothing parameter are based on the rule of thumb, cross-validation or plug-in methods. Smoothing parameter selection for distribution functions has been proposed by Altman and Leger (1995), Sarda (1993) and Bowman et al. (1998). Derumigny and Fermanian (2019) propose a cross-validation bandwidth selection procedure for the conditional Kendall’s tau. Here, we propose a plug-in pointwise bandwidth selection method for the nonparametric conditional Kendall’s tau by minimizing the overall mean squared error of the conditional tau.

The bias and variance for computing the MSE are estimated via the jackknife method based on Quenouille (1956) for bias and Tukey (1958) for variance. The procedure is an iterative process strongly related to the bootstrap resampling method proposed by Efron (1979). Actually, the jackknife is a linear approximation of the bootstrap (Abdi and Williams 2010) that entails lower computational costs and is more suitable for small data samples (Oyeyemi 2008; Efron 1982). The main steps for selecting the bandwidth for the conditional Kendall’s tau are summarized in Algorithm 1. We consider $h_0=0.9An^{-1/5}$ (Silverman 1986) as the initial bandwidth for variance estimation, where $A\!=\!min(\gamma (Z)/1.34,\ \sigma (Z))$, and $\gamma (Z)$ and $\sigma (Z)$ are the interquartile range and the standard deviation of the covariable *Z*, respectively. The initial bandwidth for the bias is taken as proposed in Gijbels et al. (2011).
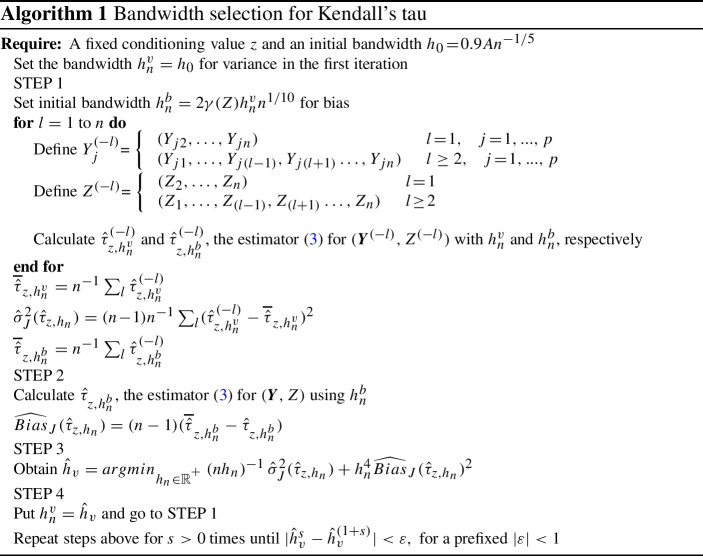


### Testing for restrictions in conditional dependence

In this section, we propose a test for linear restrictions for all null hypothesis that can be expressed as

4 $$\begin{aligned} H_0: \varvec{R}\,\varvec{\tau _z}=\varvec{r}, \end{aligned}$$

where $\tau _z=(\tau _{z_1},\ldots ,\tau _{z_m})'$ is a *m*-dimensional column vector of Kendall’s taus and $z_1,\ldots ,z_m$ are *m* deterministic conditioning values in the range of the covariable Z. Actually, $z_1,\ldots ,z_m$ are determined to be sufficiently spaced so that the subsamples used in the nonparametric estimator of the conditional Kendall’s tau for each $\{\tau _{z_\ell }\}_{\ell =1}^m$ do not overlap. $\varvec{R}$ is a $q\times m$ matrix of rank $q\le m$ and $\varvec{r}$ is a *q*-dimensional column vector where *q* is the number of restrictions to be tested. Both $\varvec{R}$ and $\varvec{r}$ are deterministic. The alternative is $H_a: \varvec{R}\,\varvec{\tau _z}\ne \varvec{r}$. The test statistic under $H_0$ is

5 $$\begin{aligned} \mathcal{J}_{n}=nh_n(\varvec{R}\,\varvec{\hat{\tau }_{_{z,h_n}}}-\varvec{r})'(\varvec{R}\varvec{V}_{\!\hat{\tau }_{_{z,h_n}}}\varvec{R}')^{-1}(\varvec{R}\,\varvec{\hat{\tau }_{_{z,h_n}}}-\varvec{r}), \end{aligned}$$

where $\varvec{V}_{\!\hat{\tau }_{_{z,h_n}}}\!\!$ is the covariance matrix of $\varvec{\hat{\tau }_{_{z,h_n}}}$, and $h_n$ is the bandwidth. The following proposition establishes the asymptotic distribution of the test statistic $\mathcal{J}_{n}$ in (5) and the asymptotic local power for local alternatives of type $H_a(\xi _n): \varvec{R}\,\varvec{\tau _z}=\varvec{r}+\xi _n\, \varvec{\varsigma }$, where $\varvec{\varsigma }$ is a $q\times 1$ nonzero deterministic column vector and $\xi _n\rightarrow 0$ as $n\rightarrow \infty$.

#### Proposition 2

Consider the same assumptions as in Proposition 1 and a set of conditioning values $\varvec{z}=(z_1,\ldots ,z_m)$, $m\!<\!n$, sufficiently spaced between them such that the subsamples used in the estimation for each $z_\ell \in \varvec{z}$ are disjoint to ensure independence. Under the null hypothesis, the $\mathcal{J}_{n}$ statistic asymptotically has a $\chi ^2$ distribution with *q* degrees of freedom.

Under local alternatives $H_a(\xi _n)$ with $\xi _n=(nh_n)^{-1/2}$, the $\mathcal{J}_{n}$ statistic is asymptotically distributed as a non-centered $\chi ^2$ distribution with *q* degrees of freedom and the noncentrality parameter $\delta _n\!=\!\varvec{\varsigma }'(\varvec{R}\varvec{V}\!_{\!\hat{\tau }_{_{z,h_n}}}\!\varvec{R}')^{-1}\!\varvec{\varsigma }$.

The limiting distributions in Proposition 2 can be obtained from the joint asymptotic normality of the multivariate Kendall’s tau conditioned to different points and Slutsky’s theorem. An outline of the proof is given in Appendix A.

The main steps for the practical implementation of the above test are presented in Algorithm 2. Without loss of generality, we consider a single bandwidth value, although it can be generalized to local bandwidth values. Note that in practice, $\varvec{V}_{\!\hat{\tau }_{_{z,h_n}}}\!\!$ must be consistently estimated. An alternative is considered in Step 2. In related papers, Gijbels et al. (2017) and Lemyre and Quessy (2017) propose different resampling procedures to test for covariate effects. We propose adapted resampling procedures to test for the hypothesis considered that will be detailed in each case.
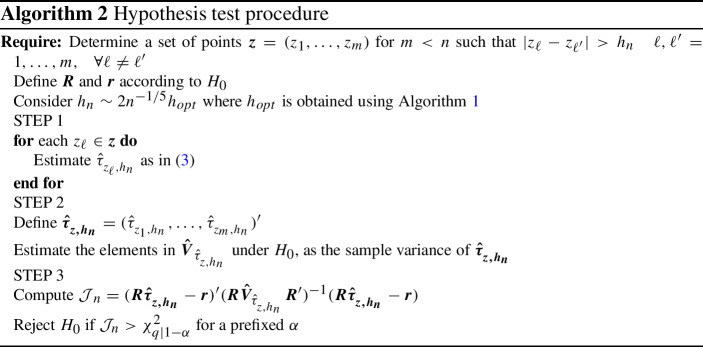


The null hypothesis in expression (4) accounts for many possible situations. In particular, it enables to test for conditionally constant dependence. Alternative tests to determine whether there are covariate effects for conditional distributions can be found in Lemyre and Quessy (2017), and for conditional copulas in Gijbels et al. (2017) and Derumigny and Fermanian (2017). Specifically, Gijbels et al. (2017) review some existing procedures purely based on conditional copula structures and introduce some nonparametric proposals using conditional Kendall’s tau.

In this particular case, $$q=m-1$$, $$\varvec{R}$$ is a $$(m-1)\times m$$ matrix with ones in the main diagonal and – 1 values in the upper diagonal, and $$\varvec{r}=\varvec{0}_{(m-1)\times 1}$$. Due to the complexity of the asymptotic variance–covariance matrix, we use a permutation procedure to estimate $$\varvec{\hat{V}}_{\!\hat{\tau }_{_{z,h_n}}}$$ under the null hypothesis: Keep *Z* fixed and obtain permuted $$\{(Y_{1i}^{b},\ldots ,Y_{pi}^{b})\}_{i=1}^n\!$$$$p-$$tuples from $$\{(Y_{1i},\ldots ,Y_{pi})\}_{i=1}^n$$ for a large number of permutations *B*. Then, with the permuted samples estimate $$\{\hat{\tau }_{_{z_\ell ,h_n}}^{b}\}_{b=1}^B$$ for each $$\ell =1,...,m$$ and compute the sample variance of the set of estimated conditional Kendall’s taus.

The statistic $$\mathcal{J}_{n}$$ can also be used to test linear restrictions across different waves. Let $$s_1$$ and $$s_2$$ be two independent samples. Then, $$\varvec{\tau _z}$$ is a $$2m\times 1$$ stacked vector accounting for the conditional dependence in the two samples, $$\varvec{\tau _z}=(\tau _{z_1}^{s_1},\ldots ,\tau _{z_m}^{s_1},\tau _{z_1}^{s_2},\ldots ,\tau _{z_m}^{s_2})= ({\varvec{\tau _z}^{s_1\ '}}, {\varvec{\tau _z}^{s_2\ '}})'$$. $$\varvec{R}=(\varvec{I}_m, -\varvec{I}_m)$$, where $$\varvec{I}$$ is the identity matrix, and $$\varvec{r}=\varvec{0}_{2m\times 1}$$. The estimated variance–covariance matrix $$\varvec{\hat{V}}_{\!\hat{\tau }_{_{z,h_n}}}$$ is now a block diagonal matrix with ($$\varvec{\hat{V}}^{s_1}_{\!\hat{\tau }_{_{z,h_n}}}, \varvec{\hat{V}}^{s_2}_{\!\hat{\tau }_{_{z,h_n}}}$$) in the diagonal and $$\varvec{\widehat{C}ov}(\varvec{\hat{\tau }_{_{z,h_n}}}^{\!\!\!\!\!\!\!\!\!\!s_1}_{ }, \varvec{\hat{\tau }_{_{z,h_n}}}^{\!\!\!\!\!\!\!\!\!\!s_2}_{ })$$ in the nondiagonal. The permutation procedure to estimate $$\varvec{\hat{V}}_{\!\hat{\tau }_{_{z,h_n}}}$$ in this context is quite different since it has to be adapted into an appropriate resampling procedure. For each sample $$s=s_1,s_2$$, a bootstrap procedure is implemented: Bootstrap $$\{(Y_{s,1i}^{b},\ldots ,Y_{s,pi}^{b},Z_{s,i}^b)\}_{i=1}^n\!$$$$(p+1)-$$tuples from $$\{(Y_{s,1i},\ldots ,Y_{s,pi},Z_{s,i})\}_{i=1}^n$$ for a sufficiently large number of times *B*. Estimate $$\hat{\tau }_{_{z_{\ell },h_n}}^{s,b}$$ for each bootstrapped sample and calculate the variances $$\hat{\sigma }^2(\hat{\tau }_{_{z_\ell ,h_n}})=(2B)^{-1} \sum _{s,b} (\hat{\tau }_{_{z_\ell ,h_n}}^{s,b}-\overline{\hat{\tau }}_{_{z_\ell ,h_n}})^2$$. Then, set $$\varvec{\hat{V}}_{\!\hat{\tau }_{_{z,h_n}}}\!\!$$ to be a diagonal matrix with size $$2m\times 2m$$ and the estimated values $$\{\hat{\sigma }^2(\hat{\tau }_{_{z_\ell ,h_n}})\}_{\ell =1}^m$$.

These two applications of the $\mathcal{J}_{n}$ statistic are implemented in the simulation study presented in the next section.

## Simulation study

### Bandwidth robustness

We consider two models to study the performance of the conditional Kendall’s tau estimator and the robustness of the bandwidth selection from Algorithm 1. For the sake of simplicity and ease of comparison, the data are generated under constant conditional dependence. In the first model (*Model L*), variables $Y_1$ and $Y_2$ depend linearly on a third variable *Z*: $Y_{1i}=7Z_i+\varepsilon _{1i}$ and $Y_{2i}=9Z_i+\varepsilon _{2i}$. In the second model (*Model NL*), the dependence of $Y_1$ and $Y_2$ on *Z* is nonlinear: $Y_{1i}=4e^{Z_i}+\varepsilon _{1i}$ and $Y_{2i}=5e^{Z_i}+\varepsilon _{2i}$. In both models, *Z* is an i.i.d variable uniformly distributed between 0 and 1 and independent from the error terms. The error terms are two i.i.d random variables such that $\varepsilon _{1i}\sim N(0,1)$, $\varepsilon _{2i}=\rho \varepsilon _{1i}+\sqrt{1-\rho ^2}\epsilon _i$ with $\epsilon _i\sim N(0,1)$ also i.i.d and independent from $\varepsilon _{1i}$, and $\rho =0, 0.75$. Note that normality implies a direct link between Pearson’s linear correlation coefficient and Kendall’s tau. Therefore, $\rho =0$ means that the dependence of $Y_1$ and $Y_2$ is fully explained by the relationship with *Z*, while $\rho =0.75$ indicates that there is an added dependency not related to *Z*. For the two models considered $S=1000$ samples of sizes $n=250, 500$, and 1000 are generated. The Epanechnikov kernel is used and the smoothing parameter for the Kendall’s tau estimator is selected via Algorithm 1.

The results (reported in the supplementary materials, Appendix B.1) show that the conditional Kendall’s tau estimator is highly sensitive to the selection of the correct smoothing parameter. As expected, for high values of the smoothing parameter, the conditional Kendall’s tau tends to an unconditional value. The smoothing parameter selection proposed in Sect. 2.1 performs quite well and the estimated conditional Kendall’s tau figures are quite close to the real conditional Kendall’s tau, regardless of the dependence structure between the variables.

### Testing for constant conditional Kendall’s tau

This section studies the size and power performance of the test proposed in Sect. 2.2. Given that the linear restriction accounts for the constant conditional Kendall’s tau among others, the proposed statistic is an alternative to the statistics proposed by Gijbels et al. (2017). We analyze the behavior of the test statistic for $H_0: \tau _{z_1}=\dots =\tau _{z_m}$, where $z_1,\dots ,z_m$ are *m* distinct conditioning values of a covariable Z. For the purpose of comparison, the $V_{n1}=n^{-1}\sum _i\left( \hat{\tau }_{_{Z_i,h_n}}\!-\!\overline{\hat{\tau }}_{\!_{Z,h_n}}\right) ^2$ statistic proposed in Gijbels et al. (2017) is considered, where $\overline{\hat{\tau }}_{\!_{Z,h_n}}=n^{-1}\sum _i \hat{\tau }_{_{Z_i,h_n}}$. Note that their statistic requires estimating Kendall’s tau at each observation of Z. Beyond bivariate models, we also study the behavior of the two test statistics when data are generated from multivariate dependence structures.

We consider the independence case and two different settings: Models based on single copulas and models based on mixture copulas. For all models, $S=1000$ samples are generated with sample sizes $n=250, 500$, and 1000. The conditioning variable *Z* is generated as an i.i.d random variable.

*Independence setting*

*Model 1:* Data are generated assuming a nonlinear specification for the marginals: $Y_{1i}=4e^{Z_i}+\varepsilon _{1i}$, $Y_{2i}=5e^{Z_i}+\varepsilon _{2i}$, where $\varepsilon _{1i},\varepsilon _{2i}$ are two independent i.i.d variables normally distributed with zero mean and unit variance. The conditioning variable is uniform between 0 and 1, and it is independent of $\varepsilon _{1i},\varepsilon _{2i}$. (This is the particular case of ‘*Model NL*’ defined in Sect. 3.1 for $\rho =0$.)

*Single copula setting*

Five cases are generated from single copula models $C(F_1\!(Y_{1i}),\!\ldots \!,F_p(Y_{pi});\!\theta )$ where $\theta$ is the dependence parameter of the copula in each model.

*Model 2:* The data-generating process comes from a bivariate Clayton copula where the marginals $\{Y_{1i}\}$ and $\{Y_{2i}\}$ are normally distributed i.i.d variables with zero mean and unit variance, and the dependence parameter is $\theta _2(Z_i)=Z_i^2/(Z_i^2+1)$ with Z uniform between 0 and 6.
*Model 3:* Data are generated from a bivariate Gumbel copula with marginals $Y_{1i}=2\sin (2\pi /3(Z_i-2)-1)+\varepsilon _{1i}$ and $Y_{2i}=\varepsilon _{2i}$, where $\{\varepsilon _{1i}\}$ and $\{\varepsilon _{2i}\}$ are two i.i.d sequences that have density $1-|x|$ on the support $[-1,1]$, the dependence parameter is given by $\theta _3(Z_i)=e^{0.5}+1$, and Z is uniform between 2 and 5. (This model is defined as ‘Model 1’ in Gijbels et al. (2017), Sect. 5.)
*Model 4:* Data are generated as in *Model 3*, but for the dependence parameter of the Gumbel copula, the function $\theta _4(Z_i)=e^{1.5-0.4Z_i}+1$ is taken. (This model is defined as ‘Model 2’ in Gijbels et al. (2017), Sect. 5.)
*Model 5:* Data come from a multivariate Clayton copula with $p=3$. The sequences $\{Y_{1i}\}$ and $\{Y_{2i}\}$ are generated as in *Model 3* and $Y_{3i}=5+3\cos (14\pi /3\,(Z_i-2)-7)+\varepsilon _{3i}$, where $\{\varepsilon _{3i}\}\sim N(0,1)$ is an i.i.d random variable. The copula dependence parameter is $\theta _5(Z_i)=\theta _3(Z_i)$, where Z is uniform between 2 and 5.
*Model 6:* The data-generating process is similar to that in *Model 5*. In this model, the marginal $\{Y_{3i}\}\sim N(0,1)$ is an i.i.d random variable, and the Clayton copula dependence parameter functional has changed to $\theta _6(Z_i)=e^{1.5+0.4Z_i}+1$.

*Mixture copula setting*

The second set, *Models* *7* to *10*, considers data generated from mixture copulas

$$\begin{aligned} C^{MIX}\!(u_1,\ldots ,u_p)\!=\!w C_a(u_1,\ldots ,u_p;\theta _{a})\!+\!(1\!-\!w) C_b(u_1,\ldots ,u_p;\theta _{b}), \end{aligned}$$

where $\theta _{r}$ is the dependence parameter for $r=a,b$ and *w* is the weight function.

*Model*7 :  In this case, bivariate Clayton and Gumbel copulas are considered for $C_a$ and $C_b$ with $w=0.3$, where marginals $\{Y_{1i}\}$ and $\{Y_{2i}\}$ are two i.i.d variables with standard normal distribution, the dependence parameters are $\theta _a(Z_i)=e^{0.5}$ and $\theta _b(Z_i)=1.2$, and Z is uniform between 0 and 6.
*Model 8:* Data come from a mixture between two bivariate Frank copulas with $w=0.3$. The marginals $Y_{1}$ and $Y_{2}$ are generated as in *Model 3*, $\theta _a(Z_i)=Z_i^3/(Z_i^3+1)$, $\theta _b(Z_i)=\theta _6(Z_i),$ and Z is uniform between 2 and 5.
*Model 9:* Multivariate Clayton and Gumbel copulas (with $p=3$) are taken as $C_a$ and $C_b$ with $w=0.7$, respectively. $Y_{1i}=2\sin (\pi \,(Z_i/3-1))+\varepsilon _{1i}$, $Y_{2i}=\varepsilon _{2i}$, and $Y_{3i}=\varepsilon _{3i}$, where $\{\varepsilon _{1i}\}$, $\{\varepsilon _{2i}\}$, and $\{\varepsilon _{3i}\}$ are three independent i.i.d sequences of normally distributed random variables with zero mean and unit variance. The dependence parameters are defined as $\theta _a(Z_i)=sin(4\pi /7)+1$ and $\theta _b(Z_i)=e^{2.5}+1$, where Z is uniform between 0 and 6.
*Model 10:* Data are generated as in *Model 8*, considering multivariate ($p=3$) versions for the Frank copulas. The third marginal $\{Y_{3i}\}$ is an i.i.d random variable with density $1-|x|$ on the support $[-1,1]$.

The rejection frequencies for each model are presented in Table 1 for levels $\alpha =1\%,5\%$, and $10\%$ and for the different sample sizes. *Models 1, 3, 5, 7*, and *9* consider a constant conditional dependence, so the null hypothesis holds. Thus, these results report the size. By contrast, for *Models 2, 4, 6, 8*, and *10*, the conditional dependence is *Z*-dependent, so the results report the power of the test. The three-column first block contains the rejection frequencies for the proposed $\mathcal{J}_{n}$ test statistic when the conditioning points are about the $5\%$ of the sample size, $\mathcal{J}_{n}^{5\%}$. This percentage of points is used because the simulation study provides optimal results in terms of size and power for that proportion at a low computational cost. Note that the conditioning points have to be sufficiently spaced so that the subsamples used in the estimation for each conditioning value are disjoint. The three-column last block shows the results for the $V_{n1}$ statistic proposed by Gijbels et al. (2017). Additional rejection frequency results for $\mathcal{J}_{n}$ when the set of conditional points are selected as $2.5\%$ and $10\%$, sufficiently spaced points of the sample ($\mathcal{J}_{n}^{2.5\%}$ and $\mathcal{J}_{n}^{10\%}$) are given in the supplementary materials, Appendix B.2. The performance of the two tests is studied in the case of unknown marginals.

**Table 1:** Rejection frequencies for simulated models (sample sizes $n=250,500, 1000$ and $S=1000$ replications)

		$\mathcal{J}_{n}^{5\%}$			$V_{n1}$		
		*n*			*n*		
	$\alpha$	250	500	1000	250	500	1000
Model 1	$1\%$	1.8	0.7	0.8	1.4	0.2	1.1
	$5\%$	5.8	4.5	4.9	6.9	4.8	3.9
	$10\%$	10.6	8.9	10.2	13.1	8.5	8.9
Model 2	$1\%$	97.2	98.7	99.6	3.7	19.1	57.0
	$5\%$	98.3	99.8	100.0	12.4	39.1	80.5
	$10\%$	99	99.8	100.0	20.1	51.3	87.5
Model 3	$1\%$	1.0	2.2	1.0	0.5	1.5	2.6
	$5\%$	4.1	6.3	4.8	4.3	6.7	10.9
	$10\%$	8.8	11.1	10.3	10.1	11.5	17.6
Model 4	$1\%$	83.7	96.4	99.4	7.3	10.6	25.5
	$5\%$	91.6	99.1	99.8	21	28.4	47.2
	$10\%$	94.5	99.5	99.9	33.3	37.7	57.7
Model 5	$1\%$	3.0	1.7	1.9	1.0	1.2	1.0
	$5\%$	6.6	4.8	5.2	7.1	5.7	5.1
	$10\%$	10.0	7.2	9.5	13.6	12.9	14.4
Model 6	$1\%$	17.6	69.3	99.3	2.9	9.2	60.9
	$5\%$	31.2	84.2	99.7	8.1	25.9	80.4
	$10\%$	40.6	89.9	99.8	14.0	36.9	86.5
Model 7	$1\%$	0.9	1.7	1.7	2.3	0.6	0.8
	$5\%$	4.4	6.9	8.1	8.1	3.9	5.6
	$10\%$	8.7	12.3	14.2	12.5	6.2	11.5
Model 8	$1\%$	6.2	22.9	56.5	11.3	16.7	34.4
	$5\%$	19.3	43.0	71.8	24.6	30.4	54.3
	$10\%$	30.1	55.7	78.6	32.7	40.8	62.5
Model 9	$1\%$	2.4	1.5	2.4	0.2	1.8	2.8
	$5\%$	7.4	6.5	5.6	1.4	10.0	8.1
	$10\%$	12.5	11.2	9.0	2.8	15.3	14.8
Model 10	$1\%$	22.1	75.5	93.5	12.7	30.4	53.7
	$5\%$	43.9	87.2	97.0	31.2	50.7	76.6
	$10\%$	56.1	91.6	97.8	44.4	62.6	84.0

The results support the idea that the proposed test statistic is appropriate for testing constant conditional dependence in bivariate and multivariate contexts. *Models 3* and *4* are also analyzed by Gijbels et al. (2017) for $n=100$ and $\alpha =0.05$. The results obtained for those models seem to be in line with the ones obtained in Gijbels et al. (2017), at least for the small sample size. Indeed, $\mathcal{J}_{n}^{5\%}$ appears to be more powerful than the $V_{n1}$ statistic in most cases, with a clear improvement in the case of mixture copulas. Note that in this case, compared to the structures studied in Gijbels et al. (2017), the competing models are mixtures between two Archimedean copulas where the dependence parameters are *Z*-dependent and the marginals are assumed to be unknown. Moreover, the performance of the test statistics is studied in larger sample sizes than in the analysis in Gijbels et al. (2017). Note also that while $\mathcal{J}_{n}^{5\%}$ statistic uses only some sufficiently spaced points of the covariable, $V_{n1}$ statistic requires estimating the conditional Kendall’s tau at every sample value of *Z*. This is not a big drawback in small sample sizes, but it entails high computational costs for large samples. In practice, this is a key issue. Moreover, $\mathcal{J}_{n}^{5\%}$ is computationally less expensive to compute than $V_{n1}$. Therefore, the results support the conclusion that the $\mathcal{J}_{n}^{5\%}$ statistic is a good alternative for testing for constant conditional dependence.

### Testing for equal conditional Kendall’s tau across samples

This section uses the $\mathcal{J}_{n}$ statistic to test for equality of the conditional Kendall’s tau across two samples $s_1$ and $s_2$ in a bivariate case. In this case, the null is $H_0: \varvec{\tau _{z}}^{s_1}=\varvec{\tau _{z}}^{s_2}$, where $\varvec{\tau _{z}}^{s}=(\tau _{z_1}^s,\dots ,\tau _{z_m}^s)$ for *m* sufficiently spaced conditioning values in the range of the covariable *Z* in the sample *s* that meet the requirements established in Sect. 3.2. Note that the conditional Kendall’s tau may change with *Z*.

In order to analyze the performance of the statistic in line with the sample size in the empirical part, we simulate two samples with $n=250$ observations under four scenarios:

*Scenario 1:* The two samples are generated as in *Model 2*.
*Scenario 2:* First and second samples are generated as in *Models 2* and *4*, respectively.
*Scenario 3:* The samples are generated as in *Model 8*.
*Scenario 4:* First and second samples are generated as in *Models 8* and *4*, respectively.

The results from *Scenarios 1* and *3* report the size of the test and those from *Scenarios 2* and *4* report its power. In all cases, 4000 replications are taken. Table 2 presents the rejection frequencies for *Scenarios 1* to *4* for different significance levels. The statistic provides adequate results in this aplication even for complicated structures such as mixtures of copulas.

**Table 2:** Rejection frequencies of $\mathcal{J}_{n}^{5\%}$ statistic for simulated scenarios (sample size $n=250$ and $S=4000$ replications)

	$\alpha =1\%$	$\alpha =5\%$	$\alpha =10\%$
Scenario 1	1.35	4.97	9.87
Scenario 2	43.32	61.32	70.92
Scenario 3	0.62	3.97	7.72
Scenario 4	73.87	87.07	91.92

## Empirical application

The European Regional Competitiveness Index (RCI) has been drawn up by the European Commission every three years since 2010^1^. It comprises more than 70 indicators for measuring the ability of regions to offer an attractive, sustainable environment for firms and residents to live and work in (Annoni and Dijkstra 2019). The final index is formed by eleven pillars grouped into three general categories: Basic, Efficiency, and Innovation. Basic comprises five pillars: Institutions (*INST*), Macroeconomic Stability, Infrastructure, Health, and Basic Education. Efficiency comprises three pillars: Higher Education (*HE*), Labor market efficiency (*L*), and Market size (*M*). Innovation also comprises three: Technological readiness (*TR*), Business Sophistication (*BS*), and Innovation (*I*). The measures of the pillars are provided as *z*-scores, and the values are such that higher means better. The data set contains the *z*-scores for the eleven pillars, the three categories, and the RCI index at regional level with 268 European regions for 2019.

It is well-known that there are links between some individual pillars and the macroeconomy. For instance, the role of higher education in economic growth has been widely analyzed in economic models (Lucas 1988). There is also a large body of literature that links innovation to economic growth (see, e.g. Furman et al. 2002, Hasan and Tucci 2010, Pradhan et al. 2016, Maradana et al. 2017). On that basis, it is helpful for political regulation and economic purposes to detect how higher education levels can affect a firm’s innovation. Haiyan et al. (2020) find evidence for China that a highly educated stock of human capital plays an important role in both the probability and the quantity of innovation at firms.

As mentioned in the Introduction, the quality of institutions is a key determinant in the prosperity of regions, and the RCI index also takes this into account. The Institutions (*INST*) pillar in the Basic category covers regional and national indicators for corruption, quality, and impartiality among others. It is based on the European Quality of Government Index (EQI), a survey on corruption and governance at a regional level within the EU conducted by the Quality Government Institute at the University of Gothenburg.^2^ As suggested in the studies collected in Dimant and Tosato (2018), there is a link between institutional quality and growth.

Based on the above ideas, we seek to detect whether low institutional quality hinders transfers between higher education and innovation results. Thus, the objective of this section is to study the link between higher education and innovation, conditional on institutional quality. Specifically, we study whether institutional quality helps to increase the bivariate and multivariate relationships between other pillars. To that end, we select some values of the *INST* variable so that they represent different institutional quality levels and we compute the conditional dependence coefficients using estimator (3). The selected values are $z_1\!=\!-1.519,\ z_2=-1.218,\ z_3= 0.160,\ z_4= 0.905$, and $z_5=1.156$ and correspond to the quantiles 0.05, 0.15, 0.5, 0.85, and 0.95 of the *INST* variable. In particular, we are interested in four different hypotheses formulated as follows:

1. There is no concordance between higher education and innovation, measured by Kendall’s tau: $H_0^{(1)}: \tau =0$. A positive tau is expected, supporting the idea that more competitive regions are linked to higher indicator scores. This hypothesis is tested using the normal asymptotic distribution of the empirical Kendall’s tau (Prokhorov 1994).
2. The concordance between higher education and innovation is fully explained by institutional quality: $H_0^{(2)}:\tau _{z}=0$, where *z* is the measure for institutional quality, the *INST* pillar. This is true if the pillars are *z*-conditional independent, which is a sufficient but not necessary condition for $\tau _{z}=0$.
3. Institutional quality does not explain any of the concordance between higher education and innovation: $H_0^{(3)}:\tau _{z}= \tau$. This means that the unconditional and conditional degrees of concordance between pillars are equal, whatever the level *z* of institutional quality.
4. The concordance between higher education and innovation might not depend on the level of quality of institutions: $H_0^{(4)}:\tau _{z_k}= \tau _{z_l}$ is tested to check whether the degree of concordance between pillars depends or not on the standard of the quality of governance, where $z_k\ne z_l$ denote two different levels of institutional quality. Rejection would be evidence of a link between pillars that varies according to institutional quality.

The fourth hypothesis is especially interesting, since its rejection provides a starting point for studying how quality of institutions makes the links between pillars stronger or weaker. Analyzing such conditional comovements is very useful for policy makers with a view to controlling the impact of their interventions.

For the sake of illustration and to provide further empirical results, we also consider the dependence between the Efficiency and Innovation groups and between higher education and the other pillars in these two groups (Labor Market Efficiency, Market Size, Business Sophistication, and Technological Readiness). First, we test for $H_0^{(1)}$ and, as expected, find that all the unconditional Kendall’s taus become significant. For the other three hypotheses, we adapt the test statistic $\mathcal{J}_n$ described in Sect. 2.2 to each hypothesis. To analyze $H_0^{(2)}$, set $\varvec{R}$ as the identity matrix $\varvec{I}_m$ and $\varvec{r}=\varvec{0}_{m\times 1}$. To test for $H_0^{(3)}$, $\varvec{R}=\varvec{I}_m$ and $\varvec{r}=\tau \cdot \varvec{1}_{m\times 1}$. Finally, for $H_0^{(4)}$, (4) as detailed in Sect. 2.2 is considered. Note that the pillars’ z-scores are not independent observations, but the adequacy of the test statistic is guaranteed by Slutsky’s and central limit theorems.

Table 3 contains the estimated unconditional Kendall’s tau in the second column and the estimated conditional coefficients in columns 3 to 7. The three-column last block summarizes the tests results.

**Table 3:** Kendall’s tau coefficients based on 2019 RCI data. The values in column 2 are the unconditional coefficients, while columns 3 to 7 show the conditional ones

Relations	$$\hat{\tau }$$	$$\hat{\tau }_{q_{0.05}}$$	$$\hat{\tau }_{q_{0.15}}$$	$$\hat{\tau }_{q_{0.5}}$$	$$\hat{\tau }_{q_{0.85}}$$	$$\hat{\tau }_{q_{0.95}}$$	$$H_0^{(2)}$$	$$H_0^{(3)}$$	$$H_0^{(4)} $$
Eff.-Inn.	0.678	0.097	0.623	0.484	0.204	0.733	***	***	***
HE-L	0.447	0.036	0.248	0.448	0.054	0.357	***	***	*
HE-M	0.197	$-$ 0.039	$-$ 0.053	$-$ 0.294	$-$ 0.009	$-$ 0.108		***	
HE-TR	0.405	0.077	0.215	0.382	0.052	$-$ 0.093	**	***	*
HE-BS	0.308	0.253	0.073	$-$ 0.056	0.154	$-$ 0.154		***	
HE-I	0.528	0.091	0.307	0.406	0.456	0.530	***	***	**
HE-L-M	0.363	0.025	0.155	0.020	0.151	0.029		***	
TR-BS-I	0.518	0.366	0.517	0.245	0.255	0.069	***	***	**
HE-L-M-TR-BS-I	0.387	0.112	0.225	0.171	0.025	0.118	***	***	

NOTE:Asterisks indicate level of significance: $$^{***} p<0.01$$, $$^{**}p<0.05$$ and $$^{*}p<0.1$$

There is a noteworthy dependence between higher education and innovation, which grows stronger as institutional quality becomes higher. For the other pillars, the results are different. An analysis of the link between *HE* and *BS* reveals that the idea that they are conditionally independent cannot be rejected. That is, for a given level of institutional quality, no dependence is found between *HE* and *BS* through the conditional Kendall’s tau. For the relationship between *HE* and *TR*, *INST* provides a further contribution for regions with medium institutional quality, which might be linked to specific regions.

For multivariate relationships, the dependence is always positive. There is a low dependence between the pillars in the Efficiency group, and the results reveal a constant effect of institutional quality among them. Pillars in the Innovation group are unconditionally more closely linked than those in the Efficiency group. Moreover, the Innovation group pillars are significantly affected by the quality of institutions and show a higher dependence for lower quality levels. The results show in fact that governance quality has a clear impact on the comovements of variables related to innovation. Nevertheless, the last row of Table 3 shows that although the quality of governance has a significant impact on the multivariate relationship between these six pillars, the effect is the same for all quality levels.

Regardless of whether or not conditional dependence is constant, the quality perception effect can vary from one period to another. To test whether the effect of institutional quality on the link between indicators is constant over a three-year period, we consider RCI index data for 2016 and compare similarities in the behavior patterns between the two stress periods. We find that in general, there are no significant changes in the dependency between pillars from 2016 to 2019, conditional on the quality of institutions. The test reveals that at the $5\%$ level, the institutional quality effect only changes significantly for the link *HE-M* over a three-year period.

## Conclusions

In this paper, we consider a nonparametric conditional copula to estimate conditional joint dependence in a multivariate context. As an overall measure of dependence, we compute a multivariate version of the rank correlation through a nonparametric conditional Kendall’s tau estimator.

Selecting the bandwidth for nonparametric estimators is an important task for densities, distributions, and regression. We derive a smoothing parameter selection procedure for the conditional Kendall’s tau and provide a simulation study to show its performance. The proposed procedure is based on the minimization of the global mean squared error, where the bias and variance terms are obtained using a jackknife approach.

A Wald-type statistic is used to test whether there is any significant linear restriction in Kendall’s tau conditional to some values of the covariate *Z*. As in Gijbels et al. (2017), the procedure enables conditional independence and constant conditional dependence to be tested for. The asymptotic distributions and the procedure for practical implementation are provided. We conduct a simulation study to analyze the size and power of the proposed test with bivariate and multivariate competing models for different sample sizes. The results show that the statistic performs well for different types of restriction, even when quite complex joint distributions are considered. Its ease of implementation, low computational cost, and wide range of applications make it a useful procedure for testing many different specifications of conditional rank correlations.

The methodology is applied to analyze the multivariate dependence between pillars in the 2019 RCI index. Specifically, we focus on the effect of the quality of institutions on the relationship between the Efficiency and Innovation pillars. The results are quite interesting and encourage further study.

First, there is a clear positive joint relationship between pillars, as expected. Second, the joint relationship between pairs such as innovation and higher education is only partially explained by the quality of institutions. Moreover, there is evidence in favor of a joint dependence that increases with the quality of institutions. In other words, the lower the quality of institutions, the weaker the link between innovation and higher education. This may be an interesting starting point for studying whether there is a causal link between the quality of institutions and the ability of regions to transfer human capital to innovation results. This goes beyond the scope of this work, but we believe that it opens up a promising research area.

## Supplementary Information

Below is the link to the electronic supplementary material.

Supplementary file 1 (pdf 1062 KB)

## Data Availability

Data are publicly available in the European Commission website.

## Footnotes

1 Data set and more details available on the European Commission website: https://ec.europa.eu/regional_policy/en/information/maps/regional_competitiveness/.

2 See https://qog.pol.gu.se/data/datadownloads/qog-eqi-data for details.
