# Supplementary material for: Testing conditional multivariate rank correlations: the effect of institutional quality on factors influencing competitiveness
Source: Test (Madr). 2022 Apr 1;31(4):931–49. doi: 10.1007/s11749-022-00806-1 (PMC8972772; doi:10.1007/s11749-022-00806-1)
Supplement: Supplementary file 1 — (pdf 1062 KB) [file 11749_2022_806_MOESM1_ESM.pdf]

Supplementary Material to "Testing conditional multivariate rank correlations. The effect of institutional quality on factors influencing competitiveness"

Jone Ascorbebeitia[a], Eva Ferreira[b], Susan Orbe[b]

Appendix A: Proofs.

This section contains the proofs of the main theoretical results stated in Section 2.

Proof of Proposition 1

To guarantee the consistency of the conditional Kendall's tau, the asymptotic bias must first be derived. Since $\sum_{i=1}^{n} w_i(z,h_n)^2 = O((nh_n)^{-1})$ when $nh_n \to \infty$, $h_n \to 0$ as $n \to \infty$, and to overcome the random denominator, the asymptotic property of the following modified estimator is derived

$$\hat{\tau}^*_{z,h_n} = \frac{1}{2^{p-1}-1}\left(2^p \sum_{i,j=1}^{n} \frac{k_{h_n}(z - Z_i)k_{h_n}(z - Z_j)}{(nh_n f(z))^2} I\{\mathbf{Y}_i < \mathbf{Y}_j\} - 1\right), \quad (1)$$

where $k_{h_n}(\cdot) = k(\cdot/h_n)$ and k is a kernel function. For technical reasons we use different bandwidths for $\hat{\tau}^*_{z,h_n}$ and $\hat{f}_{h_n^f}(z) := (nh_n^f)^{-1}\sum_{j=1}^{n} k_{h_n^f}(z - Z_j)$, h_n and h_n^f respectively, so that the following condition holds

$$\frac{E||\hat{f}_{h_n^f}(z) - f(z)||^2}{E||\hat{\tau}^*_{z,h_n} - \tau_z||^2} = o(1), \quad \text{as } h_n \to 0, n \to \infty \text{ and } nh_n \to \infty.$$

This condition of the conditional Kendall's tau establishes that $\hat{f}_{h_n^f}(z)$ converges to the real density $f(z)$ faster than the rate at which the mean squared error of the Kendall's tau $\hat{\tau}^*_{z,h_n}$ converges to zero (see Ferreira et al. (2011)).

[a]Corresponding author: Department of Quantitative Methods, University of the Basque Country UPV/EHU, Avda. Lehendakari Aguirre 83, 48015 Bilbao (Spain), email: jone.ascorbebeitia@ehu.eus

[b]Department of Quantitative Methods, University of the Basque Country UPV/EHU, Avda. Lehendakari Aguirre 83, 48015 Bilbao (Spain)

Submitted to Test

For the expected value of the $\hat{\tau}^*_{z,h_n}$ estimator it holds that

$$\begin{aligned} E\big(\hat{\tau}^*_{z,h_n}\big) &= \frac{1}{2^{p-1}-1}\left[2^p E\left[\sum_{i,j=1}^{n} \frac{k_{h_n}(z-Z_i)k_{h_n}(z-Z_j)}{(nh_n f(z))^2} I\{\mathbf{Y}_i < \mathbf{Y}_j\}\right] - 1\right] \\ &= \frac{1}{2^{p-1}-1}\left[\frac{2^p(n-1)}{n(h_n f(z))^2} E\Big(k_{h_n}(z-Z_1)k_{h_n}(z-Z_2) I\{\mathbf{Y}_1 < \mathbf{Y}_2\}\Big) - 1\right], \end{aligned}$$

where $I\{\mathbf{Y}_1 < \mathbf{Y}_2\} = I\{Y_{11} < Y_{12}, ..., Y_{p1} < Y_{p2}\}$. Applying the law of iterated expectations, the expected value above can be rewritten as follows:

$$\begin{aligned} E\big(\hat{\tau}^*_{z,h_n}\big) &= \frac{1}{2^{p-1}-1}\left[\frac{2^p(n-1)}{n(h_n f(z))^2} E\bigg(E\Big(k_{h_n}(z-Z_1)k_{h_n}(z-Z_2) I\{\mathbf{Y}_1 < \mathbf{Y}_2 | \mathbf{Y}_2, z_2\}\Big)\bigg) - 1\right] \\ &= \frac{1}{2^{p-1}-1}\left[\frac{2^p(n-1)}{n(h_n f(z))^2} E\Big(k_{h_n}(z-Z_2)\int_{\mathbb{R}} k_{h_n}(z-s) f(s) F_s(\mathbf{Y}_2) ds\Big) - 1\right] \\ &= \frac{1}{2^{p-1}-1}\left[\frac{2^p(n-1)}{n h_n f(z)^2} E\Big(k_{h_n}(z-Z_2)\int_{-1}^{1} k(u) f(z-uh_n) F_{z-uh_n}(\mathbf{Y}_2) du\Big) - 1\right] \\ &= \frac{1}{2^{p-1}-1}\left[\frac{2^p(n-1)}{n h_n f(z)^2} E\bigg(k_{h_n}(z-Z_2)\Big(F_z(\mathbf{Y}_2) f(z) + \frac{h_n^2 c_k}{2}\Big(F_z(\mathbf{Y}_2) f''(z)\right. \\ &\quad \left. + 2F'_z(\mathbf{Y}_2) f'(z) + F''_z(\mathbf{Y}_2) f(z)\Big) + o\left(h_n^2\right)\Big)\bigg) - 1\right]. \end{aligned}$$

Applying expectations again, the above expression is

$$\begin{aligned} E\big(\hat{\tau}^*_{z,h_n}\big) &= \tau_z + \frac{2^{p-1} h_n^2 c_k}{(2^{p-1}-1) f(z)} \int_{\mathbb{R}^p} F_z(\boldsymbol{y})\Big(f_z(\boldsymbol{y}) f''(z) + 2f'_z(\boldsymbol{y}) f'(z) + f''_z(\boldsymbol{y}) f(z)\Big) \\ &\quad + f_z(\boldsymbol{y})\Big(F_z(\boldsymbol{y}) f''(z) + 2F'_z(\boldsymbol{y}) f'(z) + F''_z(\boldsymbol{y}) f(z)\Big) d\boldsymbol{y} + o(h_n^2) \\ &:= \tau_z + \frac{2^{p-1} h_n^2 c_k}{(2^{p-1}-1) f(z)} \int_{\mathbb{R}^p} \Big(F_z(\boldsymbol{y}) g(f_z(\boldsymbol{y})) + f_z(\boldsymbol{y}) g(F_z(\boldsymbol{y}))\Big) d\boldsymbol{y} + o(h_n^2), \end{aligned}$$

where $g(r_z(\boldsymbol{y})) = \Big(r_z(\boldsymbol{y}) f''(z) + 2r'_z(\boldsymbol{y}) f'(z) + r''_z(\boldsymbol{y}) f(z)\Big)$. As a consequence, the asymptotic bias of the multivariate estimator $\hat{\tau}^*_{z,h_n}$ is given by $Bias(\hat{\tau}^*_{z,h_n}) = 2^{p-1} h_n^2 c_k ((2^{p-1}-1) f(z))^{-1} \times \int_{\mathbb{R}^p}\Big(F_z(\boldsymbol{y}) g(f_z(\boldsymbol{y})) + f_z(\boldsymbol{y}) g(F_z(\boldsymbol{y}))\Big) d\boldsymbol{y} + o(h_n^2)$. Since $\sum_{i=1}^n w_i(z, h_n)^2 = O((nh_n)^{-1})$, the result still holds for $\hat{\tau}_{z,h_n}$.

Then, we derive the asymptotic distribution of the $\hat{\tau}_{z,h_n}$ estimator through the convergence of the multivariate copula in a similar way to that already used for conditional copulas by Veraverbeke et al. (2011) and for right-censored length-biased data by Rabhi and Bouezmarni (2019), both in the bivariate case. In fact, it holds that $\int_{I^p} \hat{C}_{z,h_n}(\boldsymbol{u}) d\hat{C}_{z,h_n}(\boldsymbol{u}) =$

$(nh_n)^{-1}\sum_{j=1}^n w_j(z,h_n)\hat{C}_{z,h_n}(\hat{\boldsymbol{F}}_{z,h_n}(\mathbf{Y}_j))$ provided that the left-hand side is the estimated mean of $\hat{C}_{z,h_n}(\boldsymbol{u})$. Thus, substituting the copula estimator $\hat{C}_{z,h_n}(\boldsymbol{u})$ in the latter expression leads to $\sum_{i,j=1}^n w_j(z,h_n)w_i(z,h_n)I\{\mathbf{Y}_i < \mathbf{Y}_j\} + \sum_{j=1}^n w_j(z,h_n)^2$. Hence, assuming that $\sum_{j=1}^n w_j(z,h_n)^2 = O\left((nh_n)^{-1}\right)$ holds, $\hat{\tau}_{z,h_n}$ can be written as

$$\begin{aligned}\hat{\tau}_{z,h_n} &= \frac{1}{2^{p-1}-1}\Big(\frac{2^p}{1-\sum_{j=1}^n w_j(z,h_n)^2}\int_{I^p}\hat{C}_{z,h_n}d\hat{C}_{z,h_n} - \frac{1+(2^p-1)\sum_{j=1}^n w_j(z,h_n)^2}{1-\sum_{j=1}^n w_j(z,h_n)^2}\Big)\\ &= \frac{1}{2^{p-1}-1}\Big(2^p\int_{I^p}\hat{C}_{z,h_n}d\hat{C}_{z,h_n} - 1\Big) + O\left(\frac{1}{nh_n}\right).\end{aligned}$$

Since Kendall's tau can be written as a functional of the copula, we define the map $\phi : C_z \to (2^{p-1}-1)^{-1}(2^p\int_{I^p} C_z dC_z - 1)$. In a similar way to Lemma 1 in Veraverbeke et al. (2011), ϕ is Hadamard differentiable at C_z tangentially to the set of functions on $[0,1]^p$ and its derivative is given by

$$\phi'(\xi) = \frac{2^p}{2^{p-1}-1}\left(\int_{I^p} C_z d\xi + \int_{I^p}\xi dC_z\right).$$

Hence, assuming $h_n = o(n^{-1/5})$, the delta method establishes that the asymptotic distribution of $\hat{\tau}_{z,h_n}$ is

$$(nh_n)^{1/2}(\hat{\tau}_{z,h_n} - \tau_z) = (nh_n)^{1/2}(\phi(\hat{C}_{z,h_n}) - \phi(C_z)) \to \phi'(C_z^L),$$

where C_z^L is the limiting distribution of $(nh_n)^{-1/2}(\hat{C}_{z,h_n} - C_z)$ so that the variance for $\hat{\tau}_{z,h_n}$ is determined by $\sigma^2(\phi'(C_z^L))$. Note that $h_n = o(n^{-1/5})$ is required in the weak convergence of the empirical copula as well as to remove the conditional Kendall's tau estimator's bias.

Proof of Proposition 2

To derive the asymptotic distribution of the $\mathcal{J}_n$ statistic we use the joint asymptotic normality of the conditional Kendall's tau given a set of covariate points.

Derumigny and Fermanian (2019) derive the asymptotic distribution of the conditional Kendall's tau given a vector of covariates in a bivariate context. The generalization to our multivariate context for a unidimensional covariate can be obtained following same steps.

Proposition A.1. *Let $\boldsymbol{z} = (z_1, ..., z_m)'$ be m deterministic different points. Then, under assumptions A1 to A4,*

$$(nh_n)^{1/2}(\hat{\boldsymbol{\tau}}_{\boldsymbol{z},\boldsymbol{h_n}} - \boldsymbol{\tau}_{\boldsymbol{z}}) \xrightarrow{d} N(0, \boldsymbol{V}_{\hat{\tau}_{z,h_n}}),$$

where $\boldsymbol{V}_{\hat{\tau}_{z,h_n}}$ *is a matrix whose elements are defined by*

$$[\boldsymbol{V}_{\hat{\tau}_{z,h_n}}]_{\ell s} = 4d_k f(z_\ell)^{-1} I\{\ell = s\} \Bigg(E\Big(g(\mathbf{Y}_1, \boldsymbol{Y})g(\mathbf{Y}_2, \boldsymbol{Y})|z_\ell\Big) - \tau^2_{z_\ell} \Bigg)$$

for every $1 \leq \ell, s \leq m$*,* $g(\mathbf{Y}_i, \boldsymbol{Y}) = (2^{p-1}-1)^{-1}(2^p I\{\mathbf{Y}_i < \boldsymbol{Y}\}-1)$ *and* $(\mathbf{Y}_1, \boldsymbol{Z}_1)$*,* $(\mathbf{Y}_2, \boldsymbol{Z}_2)$ *independent versions of* $(\boldsymbol{Y}, \boldsymbol{Z})$*.*

Proof of Proposition A.1.

Let $\hat{\tau}_{z,h_n}$ the conditional Kendall's tau estimator defined by (3) and define $g(\mathbf{Y}_i, \mathbf{Y}_j) = (2^{p-1}-1)^{-1}(2^p I\{\mathbf{Y}_i < \mathbf{Y}_j\}-1)$, so that $\hat{\tau}_{z,h_n}$ is a smoothed estimator of $E\big(g(\mathbf{Y}_1, \mathbf{Y}_2)|z\big)$. Note that $\hat{\tau}_{z,h_n}$ is a multivariate version of the bivariate $\tilde{\tau}_z$ estimator defined by Derumigny and Fermanian (2019), so Proposition A.1. can be proved using same arguments to those used to prove Proposition 9.

Since Kendall's tau is a second order U-statistic, the joint distribution of $\hat{\boldsymbol{\tau}}_{\boldsymbol{z},\boldsymbol{h_n}}$ at several conditioning points $\{z_\ell\}_{\ell=1}^m$ is studied based on U-statistics theory. Let us define the function $g^*(\mathbf{Y}_i, \mathbf{Y}_j) = (g(\mathbf{Y}_i, \mathbf{Y}_j) + g(\mathbf{Y}_j, \mathbf{Y}_i))/2$. Thus, the multivariate conditional Kendall's tau estimator can be expressed as $\hat{\tau}_{z_\ell,h_n} = U_{n,\ell}(g^*)/U_{n,\ell}(1)$, where for any bounded measurable function $g : \mathbb{R}^{2p} \to \mathbb{R}$,

$$U_{n,\ell}(g) = \frac{1}{n(n-1)E(k_{h_n}(z_\ell - Z))^2} \sum_{i,j=1}^{n} k_{h_n}(z_\ell - Z_i) k_{h_n}(z_\ell - Z_j) g(\mathbf{Y}_i, \mathbf{Y}_j).$$

Then, the asymptotic normality of $\hat{\tau}_{z_\ell,h_n}$ can be established through the limiting distribution of $U_{n,\ell}(g^*)$. Note that $E(U_{n,\ell}(g^*)) = \tau_{z,\ell} + o((nh_n)^{-1/2})$ and $E(U_{n,\ell}(1)) = 1 + o((nh_n)^{-1/2})$ also hold for the multivariate case. Taking into account the Hájek projection of $\{U_{n,\ell}(g)\}_{\ell=1}^m$ statistics for every g function defined above and a slightly adapted version of Lemma 17 in Derumigny and Fermanian (2019) for such functions, it can be established that

$$(nh_n)^{1/2} \Bigg(\Big(\boldsymbol{U_n}(g^*) - \boldsymbol{\tau_z} \Big), \Big(\boldsymbol{U_n}(1) - \mathbf{1}_{m\times 1} \Big) \Bigg) \xrightarrow{d} N\Big(0, \Sigma^*\Big),$$

where $\boldsymbol{U_n}(g) = (U_{n,1}(g), ..., U_{n,m}(g))$. Σ^* is a block diagonal matrix with $(\Sigma_{(g^*,g^*)}, \Sigma_{(1,1)})$ in the diagonal, $\Sigma_{(g^*,1)}$ in the nondiagonal, and elements

$$\Big[\Sigma_{(g_1,g_2)}\Big]_{\ell,s} = 4d_k f(z_\ell)^{-1} I\{z_\ell = z_s\} \int g_1(\boldsymbol{y_1}, \boldsymbol{y}) g_2(\boldsymbol{y_2}, \boldsymbol{y}) f_{z_\ell}(\boldsymbol{y}) f_{z_\ell}(\boldsymbol{y_1}) f_{z_\ell}(\boldsymbol{y_2}) d\boldsymbol{y} d\boldsymbol{y_1} d\boldsymbol{y_2},$$

for $g_1, g_2 = g^*, 1$ and $\ell, s = 1, ..., m$.

Given the nature of the conditional Kendall's tau as a functional of U-statistics, we define $\phi : (\boldsymbol{U_n}(g^*), \boldsymbol{U_n}(1)) \longrightarrow (\boldsymbol{U_n}(g^*)/\boldsymbol{U_n}(1)) = (U_{n,\ell}(g^*)/U_{n,\ell}(1))_{\ell=1,...,m}$, where its derivative is a $m\times 2m$ order Jacobian matrix $J_\phi(\boldsymbol{x}, \boldsymbol{y}) = [diag(y_1^{-1}, ..., y_m^{-1}), -diag(x_1 y_1^{-2}, ..., x_m y_m^{-2})]$. Hence, using the delta method over the map ϕ, it can be established that the joint limiting distribution of $\hat{\boldsymbol{\tau}}_{\boldsymbol{z},\boldsymbol{h_n}}$ is

$$(nh_n)^{1/2}(\hat{\boldsymbol{\tau}}_{\boldsymbol{z},\boldsymbol{h_n}} - \boldsymbol{\tau_z}) \xrightarrow{d} N(0, \boldsymbol{V}_{\hat{\tau}_{z,h_n}}),$$

where $\boldsymbol{V}_{\hat{\tau}_{z,h_n}} = J_\phi(\boldsymbol{\tau_z}, \mathbf{1}_{m\times 1})\Sigma^* J_\phi(\boldsymbol{\tau_z}, \mathbf{1}_{m\times 1})'$ and $J_\phi(\boldsymbol{\tau_z}, \mathbf{1}_{m\times 1}) = [\boldsymbol{I}_m, -diag(\boldsymbol{\tau_z})]$. Substituting the corresponding expressions and taking into account that all $\{z_\ell\}_{\ell=1}^m$ are different, $\boldsymbol{V}_{\hat{\tau}_{z,h_n}}$ is a covariance matrix whose elements are given by

$$\Big[\boldsymbol{V}_{\hat{\tau}_{z,h_n}}\Big]_{\ell,s} = 4d_k f(z_\ell)^{-1} I\{\ell = s\}\Big(E_{z_\ell}\big[g^*(\boldsymbol{Y_1}, \boldsymbol{Y})g^*(\boldsymbol{Y_2}, \boldsymbol{Y})\big] - \tau_{z_\ell}^2\Big). \qquad \blacksquare$$

To establish the limiting distribution of the test statistic, consider the asymptotic normality of the process $(nh_n)^{1/2}(\hat{\boldsymbol{\tau}}_{\boldsymbol{z},\boldsymbol{h_n}} - \boldsymbol{\tau_z})$ given by Proposition A.1., Slutsky's theorem, and the properties of the normal distribution. Hence, $(nh_n)^{1/2}(\boldsymbol{R}\hat{\boldsymbol{\tau}}_{\boldsymbol{z},\boldsymbol{h_n}} - \boldsymbol{r}) \xrightarrow{d} N(0, \boldsymbol{R}\boldsymbol{V}_{\hat{\tau}_{z,h_n}}\boldsymbol{R}')$. Since $\boldsymbol{R}$ is of full rank and $\boldsymbol{V}_{\hat{\tau}_{z,h_n}}$ is positive definite, $\boldsymbol{R}\boldsymbol{V}_{\hat{\tau}_{z,h_n}}\boldsymbol{R}'$ is invertible. Therefore, we derive

$$\mathcal{J}_n = nh_n(\boldsymbol{R}\hat{\boldsymbol{\tau}}_{\boldsymbol{z},\boldsymbol{h_n}} - \boldsymbol{r})'(\boldsymbol{R}\boldsymbol{V}_{\hat{\tau}_{z,h_n}}\boldsymbol{R}')^{-1}(\boldsymbol{R}\hat{\boldsymbol{\tau}}_{\boldsymbol{z},\boldsymbol{h_n}} - \boldsymbol{r}) \xrightarrow{d} \chi_q^2.$$

The asymptotic power of the test tends to the unity when $h_n \to 0$ and $nh_n \to \infty$ as $n \to \infty$, as long as the alternative hypothesis holds. Note that if the linear restriction is getting closer and closer to the null hypothesis as the sample size increases, the power may not converge to unity. Therefore, to analyze the asymptotic power of the test, we consider local alternatives subject to Pitman sequences such that $H_a : \boldsymbol{R}\boldsymbol{\tau_z} = \boldsymbol{r} + (nh_n)^{-1/2}\boldsymbol{\varsigma}$. We remark that the convergence rate considered for the Pitman sequences is in line with the rates considered in the sequences of local alternatives for the tests based on U-statistics (see Zheng, 1996). The technique of using Pitman drifts to study the asymptotic power of t-ratio and Wald type statistics is also common in regression settings (see Hayashi, 2000).

Under these type of local alternatives, $(nh_n)^{1/2}(\boldsymbol{R}\hat{\boldsymbol{\tau}}_{\boldsymbol{z},\boldsymbol{h_n}} - \boldsymbol{R}\boldsymbol{\tau_z}) = (nh_n)^{1/2}(\boldsymbol{R}\hat{\boldsymbol{\tau}}_{\boldsymbol{z},\boldsymbol{h_n}} - \boldsymbol{r}) - \boldsymbol{\varsigma}$, so that $(nh_n)^{1/2}(\boldsymbol{R}\hat{\boldsymbol{\tau}}_{\boldsymbol{z},\boldsymbol{h_n}} - \boldsymbol{r}) \xrightarrow{d} N(\boldsymbol{\varsigma}, \boldsymbol{R}\boldsymbol{V}_{\hat{\tau}_{z,h_n}}\boldsymbol{R}')$ holds. Provided that the latter

process has a noncentered normal distribution, the $\mathcal{J}_n$ statistic is distributed according to a noncentered χ^2 distribution with q degrees of freedom and a noncentrality parameter $\delta_n = \boldsymbol{\varsigma}'(\boldsymbol{R}\boldsymbol{V}_{\hat{\tau}_{z,h_n}}\boldsymbol{R}')^{-1}\boldsymbol{\varsigma}$.

Appendix B: Additional results

This section includes additional results to Section 3.

Appendix B.1: Bandwidth robustness

Figs. B.1 and B.2 show the estimated conditional Kendall's tau trend (solid black line) and its %95 pointwise confidence interval (grey shaded) for each model simulated and a range of bandwidth values. Results are shown for sample size $n = 1000$ (results for $n = 250, 500$ are available upon request). The asterisk indicates the estimated Kendall's tau value for the optimal bandwidth selected by the proposed algorithm. The figures in the first row are for *Model L*, where the dependence between variables relies on the conditional variable Z, and those in the second row are for *Model NL*, where the dependence goes beyond the conditional variable. For the sake of illustration, each row shows the results obtained conditional on three different values of Z. Quantiles $q_{0.25}$, $q_{0.50}$, and $q_{0.75}$ of variable Z have been chosen as indicators of low, medium, and high levels of the covariable. Horizontal dashed and dotted-dashed lines represent the real conditional and unconditional Kendall's tau respectively.

Appendix B.2: Testing for conditional Kendall's tau

Table B.1 shows the rejection frequencies of the $\mathcal{J}_n$ test for *Models 1-10*. The results for levels $\alpha = 1\%, 5\%$, and 10% by rows and different sample sizes by columns are given for all the models. The 3-column first block contains the rejection frequencies when the conditional points are selected as the 2.5% of sufficiently spaced points of the sample, $\mathcal{J}_n^{2.5\%}$, while the 3-column second block shows those for conditional points selected as the 10% points of the sample, $\mathcal{J}_n^{10\%}$.

Conditional Kendall's tau and 95% confidence interval for *Model L* and $\rho = 0$.

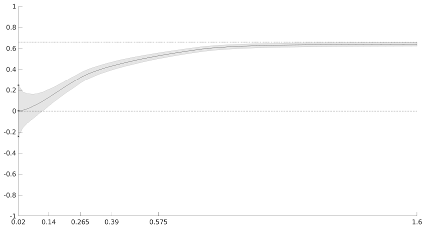

(a) $z = q_{0.25}(Z)$

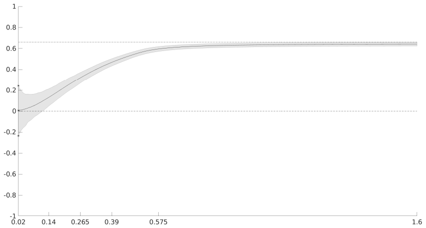

(b) $z = q_{0.50}(Z)$

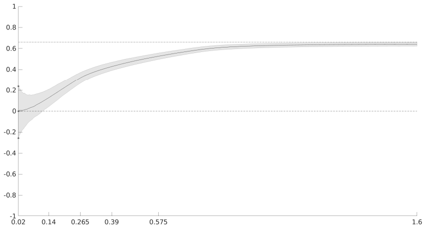

(c) $z = q_{0.75}(Z)$

Conditional Kendall's tau and 95% confidence interval for *Model L* and $\rho = 0.75$.

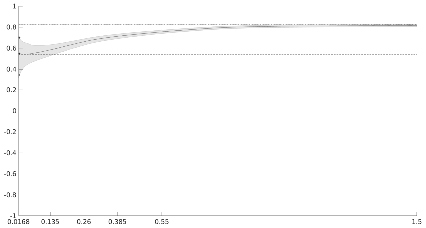

(d) $z = q_{0.25}(Z)$

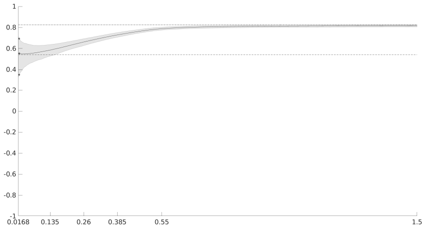

(e) $z = q_{0.50}(Z)$

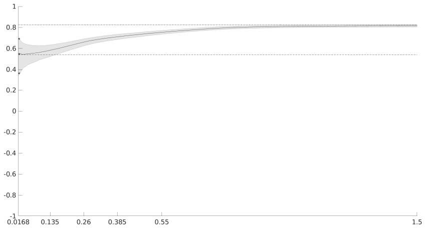

(f) $z = q_{0.75}(Z)$

Fig. B.1 Estimated Kendall's tau (solid black line) conditional on quantiles (a) 0.25, (b) 0.50, and (c) 0.75 of Z with 95% pointwise confidence interval (grey shaded) for variables generated with *Model L*, where dependence is linear and $\rho = 0, 0.75$. The asterisk represents the estimated Kendall's tau for the optimal bandwidth. Horizontal dashed and dotted-dashed lines represent the real conditional and unconditional Kendall's tau respectively. The X-axis shows the range of bandwidths.

Conditional Kendall's tau and 95% confidence interval for *Model NL* and $\rho = 0$.

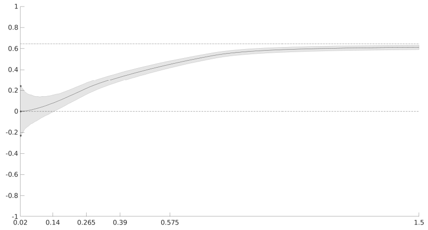

(a) $z = q_{0.25}(Z)$

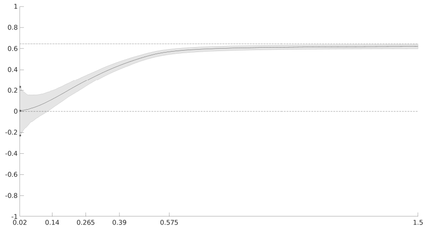

(b) $z = q_{0.50}(Z)$

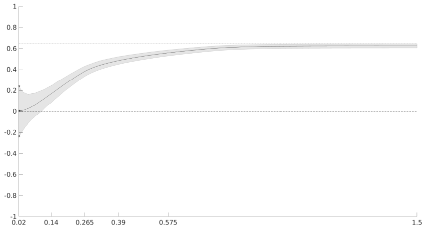

(c) $z = q_{0.75}(Z)$

Conditional Kendall's tau and 95% confidence interval for *Model NL* and $\rho = 0.75$.

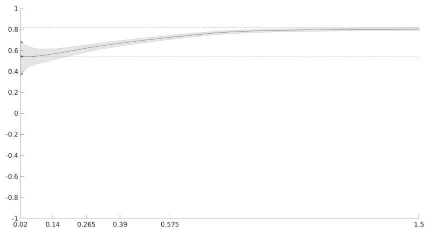

(d) $z = q_{0.25}(Z)$

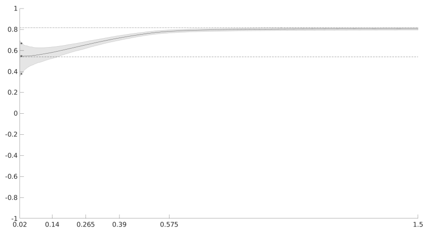

(e) $z = q_{0.50}(Z)$

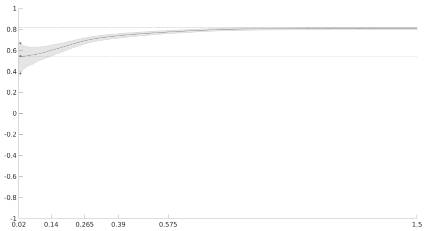

(f) $z = q_{0.75}(Z)$

Fig. B.2 Estimated Kendall's tau (solid black line) conditional on quantiles (a) 0.25, (b) 0.50, and (c) 0.75 of Z with 95% pointwise confidence interval (grey shaded) for variables generated with *Model NL*, where dependence is nonlinear and $\rho = 0, 0.75$. The asterisk represents the estimated Kendall's tau for the optimal bandwidth. Horizontal dashed and dotted-dashed lines represent the real conditional and unconditional Kendall's tau respectively. The X-axis shows the range of bandwidths.

References

Derumigny A, Fermanian JD (2019) On kernel-based estimation of conditional Kendall's tau: Finite-distance bounds and asymptotic behavior. Depend Model 7:292–321

Ferreira E, Gil-Bazo J, Orbe S (2011) Conditional beta pricing models: A nonparametric approach. J Bank Finance 35:3362–3382

Hayashi F (2000) Econometrics. Princeton University Press, Princeton

Rabhi Y, Bouezmarni T (2019) Nonparametric inference for copulas and measures of dependence under length-biased sampling and informative censoring. J Am Stat Assoc 00:1–11

Veraverbeke N, Omelka M, Gijbels I (2011) Estimation of a conditional copula and association measures. Scand J Stat 38:766–780

Zheng JX (1996) A consistent test of functional form via nonparametric estimation techniques. J Econom 75(2):263–289

Table B.1 Rejection frequencies for simulated models ($S = 1000$ replications).

		$\mathcal{J}_n^{2.5\%}$			$\mathcal{J}_n^{10\%}$		
			n			n	
	α	250	500	1000	250	500	1000
Model 1	1%	1.1	1.0	1.3	1.6	2.3	3.0
	5%	3.7	5.2	4.3	6.2	7.2	10.3
	10%	7.2	10.1	9.9	11.4	13.1	15.6
Model 2	1%	69.0	83.5	98.4	99.8	99.7	100.0
	5%	81.5	91.8	99.7	99.8	99.9	100.0
	10%	87.0	95.4	99.8	99.9	100.0	100.0
Model 3	1%	1.4	1.5	0.5	1.2	5.2	3.9
	5%	6.0	6.2	4.6	4.6	10.6	8.9
	10%	11.2	10.3	10.7	9.2	14.9	14.9
Model 4	1%	45.6	73.7	97.2	96.9	99.3	99.7
	5%	62.8	85.6	99	98.4	99.8	99.9
	10%	69.9	90.1	99.4	98.7	99.9	100.0
Model 5	1%	1.7	1.5	2.1	1.8	1.8	1.6
	5%	5.8	4.7	6.9	5.4	4	5.1
	10%	10.2	9.2	10.7	9.5	6.9	8.6
Model 6	1%	9.7	38.8	97.2	31.5	83.4	99.7
	5%	19.0	59.1	99.4	46.8	90.5	99.8
	10%	26.8	70.5	99.8	57.9	92.9	99.9
Model 7	1%	2.5	1.7	1.8	2.0	5.6	6.2
	5%	7.4	5.4	7.5	6.6	12.9	13.9
	10%	13.2	10.8	14.6	13.0	17.5	19.5
Model 8	1%	4.3	11.7	39.0	11.7	39.6	70.0
	5%	13.3	26.3	59.1	27.1	55.7	78.9
	10%	20.7	39.3	71.5	36.4	64.6	82.6
Model 9	1%	4.8	1.0	1.1	3.6	4.3	5.2
	5%	11.6	4.7	3.6	11.3	10.3	9.2
	10%	17.3	9.8	6.9	16.4	15.1	12.7
Model 10	1%	11.6	45.3	85.9	45.6	86.4	96.8
	5%	26.4	66.4	93.1	67.4	93.0	98.1
	10%	37.1	76.4	95.5	77.2	95.5	98.5
